# Supplementary material for: Trimannose-coupled antimiR-21 for macrophage-targeted inhalation treatment of acute inflammatory lung damage
Source: Nat Commun. 2023 Jul 28;14:4564. doi: 10.1038/s41467-023-40185-1 (PMC10382532; doi:10.1038/s41467-023-40185-1)
Supplement: Supplementary file 1 — Supplementary Information [file 41467_2023_40185_MOESM1_ESM.pdf]

Supplementary Information for

Trimannose-coupled antimiR-21 for macrophage-targeted inhalation treatment of acute inflammatory lung damage

Christina Beck[#1,2], Deepak Ramanujam[#1,2], Paula Vaccarello[1], Florenc Widenmeyer[1], Martin Feuerherd[3], Cho-Chin Cheng[3], Anton Bomhard[1], Tatiana Abikeeva[1], Julia Schaedler[4], Jan Peter Sperhake[4], Matthias Graw[5], Seyer Safi[6], Hans Hoffmann[6], Claudia Staab-Weijnitz[7], Roland Rad[8], Ulrike Protzer[3,9], Thomas Frischmuth[10], Stefan Engelhardt[1,2*]

[1] Institute of Pharmacology and Toxicology, Technical University of Munich (TUM), Munich, Germany

[2] DZHK (German Centre for Cardiovascular Research), partner site Munich Heart Alliance, Germany

[3] Institute of Virology, Helmholtz Center Munich, Technical University of Munich (TUM), School of Medicine, Munich, Germany

[4] Department of Legal Medicine, University Medical Center Hamburg-Eppendorf, Hamburg, Germany

[5] Department of Legal Medicine, Ludwig-Maximilians University, Munich, Germany

[6] Division of Thoracic Surgery, Klinikum rechts der Isar, Technical University of Munich, Munich, Germany

[7] Comprehensive Pneumology Center, Institute of Lung Biology and Disease, Helmholtz Center Munich, Member of the German Center of Lung Research (DZL), Munich, Germany

[8] Institute of Molecular Oncology and Functional Genomics, Translatum Cancer Center, School of Medicine, Technical University of Munich, Munich, Germany.

[9] German Center for Infection Research (DZIF), Munich Partner Site, Neuherberg, Germany

[10] Baseclick GmbH, Neuried, Germany

[#] contributed equally

* Corresponding author: Stefan.engelhardt@tum.de

This pdf file includes:

Figs. S1-18

Supplementary Table 1

Supplementary Table 2

Supplementary figures:

Fig. S1 | Increased expression and activity of miR-21 in COVID-19 lungs.

Fig. S2 | MiR-21 is highly abundant and enriched in pulmonary macrophages isolated from wild type mouse lungs.

Fig. S3 | Comparative analysis of bleomycin and COVID single cell datasets.

Fig. S4 | Characterization of mice with macrophage-specific deletion of miR-21.

Fig. S5 | *Mrc1* is expressed in pulmonary macrophages in mice and humans.

Fig. S6 | Feature plots showing expression of other C-type lectins (Cd209f, Clec4d, Clec4e and Clec5a) in mouse lungs after bleomycin-induced lung injury.

Fig. S7 | Chemical structure of trimannose-conjugated LNA-antimiR-21 (RCS-21) prepared using conventional chemistry.

Fig. S8 | Mannose saccharides included in *in silico* binding studies to CRD4 domain of MRC1.

Fig. S9 | Assessment of RCS-21-FAM in liver, kidney, heart and spleen.

Fig. S10| RCS-21 inhalation study.

Fig. S11 | Single cell sequencing of RCS-21-treated mouse lungs after acute lung injury.

Fig. S12 | Further sub-clustering of the macrophage populations shows a decrease in recruited macrophages after RCS-21 treatment in bleomycin mice.

Fig. S13 | Gene ontology analysis of transcriptomes of alveolar macrophage cluster.

Fig. S14 | Expression of extracellular matrix-related genes in fibroblast cluster.

Fig. S15 | Representative macrophage staining in hPCLS.

Fig. S16 | hPCLS were treated with FAM-labelled RCS-21.

Fig. S17 | Increased miR-21 activity in human precision cut lung slices (hPCLS) 24 hours after infection with SARS-Cov-2 (Omicron).

Fig. S18 | Viral load in SARS-CoV-2 infected and infected + RCS-21 slices.

Supplementary Table 1 | Donor metadata (post mortem study)

Supplementary Table 2 | Donor metadata (hPCLS study)

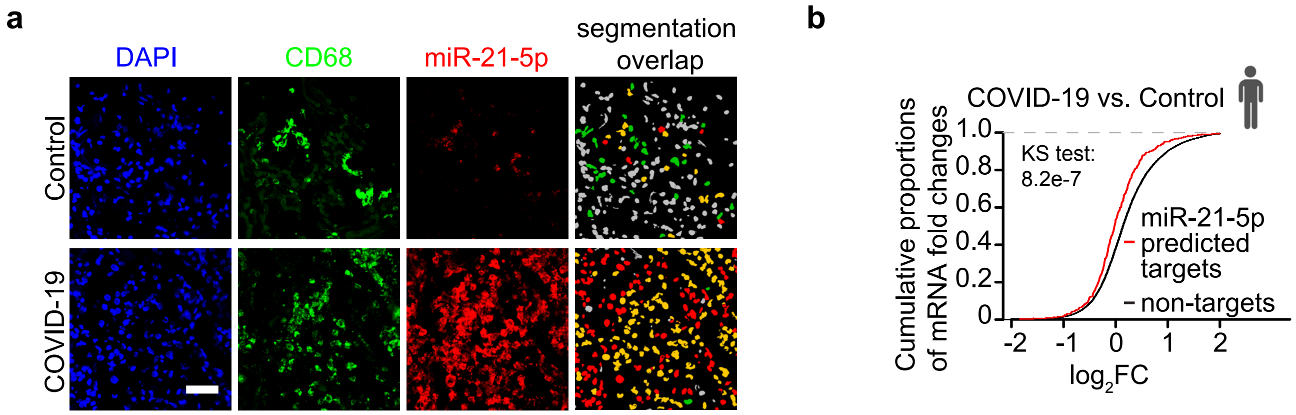

Fig. S1 | Increased expression and activity of miR-21 in COVID-19 lungs. a, Representative staining for hsa-miR-21-5p (red) and a macrophage marker CD68 (green) in control and COVID-19 lungs. Nuclei were stained with DAPI (blue). Segmentation overlap image was obtained using 'MetaMorph' Multi-Wave-length Cell Scoring application module. Masks shown define CD68 staining (green), miR-21-5p staining (red), and DAPI staining (grey). Scale bar represents 50 µm; Control n=3, COVID-19 n=3. **b**, Cumulative distribution curves represent miR-21 activity. Leftward shift of miR-21 targets (red) indicates increased miR-21 activity (COVID-19 vs control); Control n=12, COVID-19 n=14. Statistical analysis was performed using a two-sided Kolmogorov-Smirnov (KS) test. Source data are provided as a Source Data file.

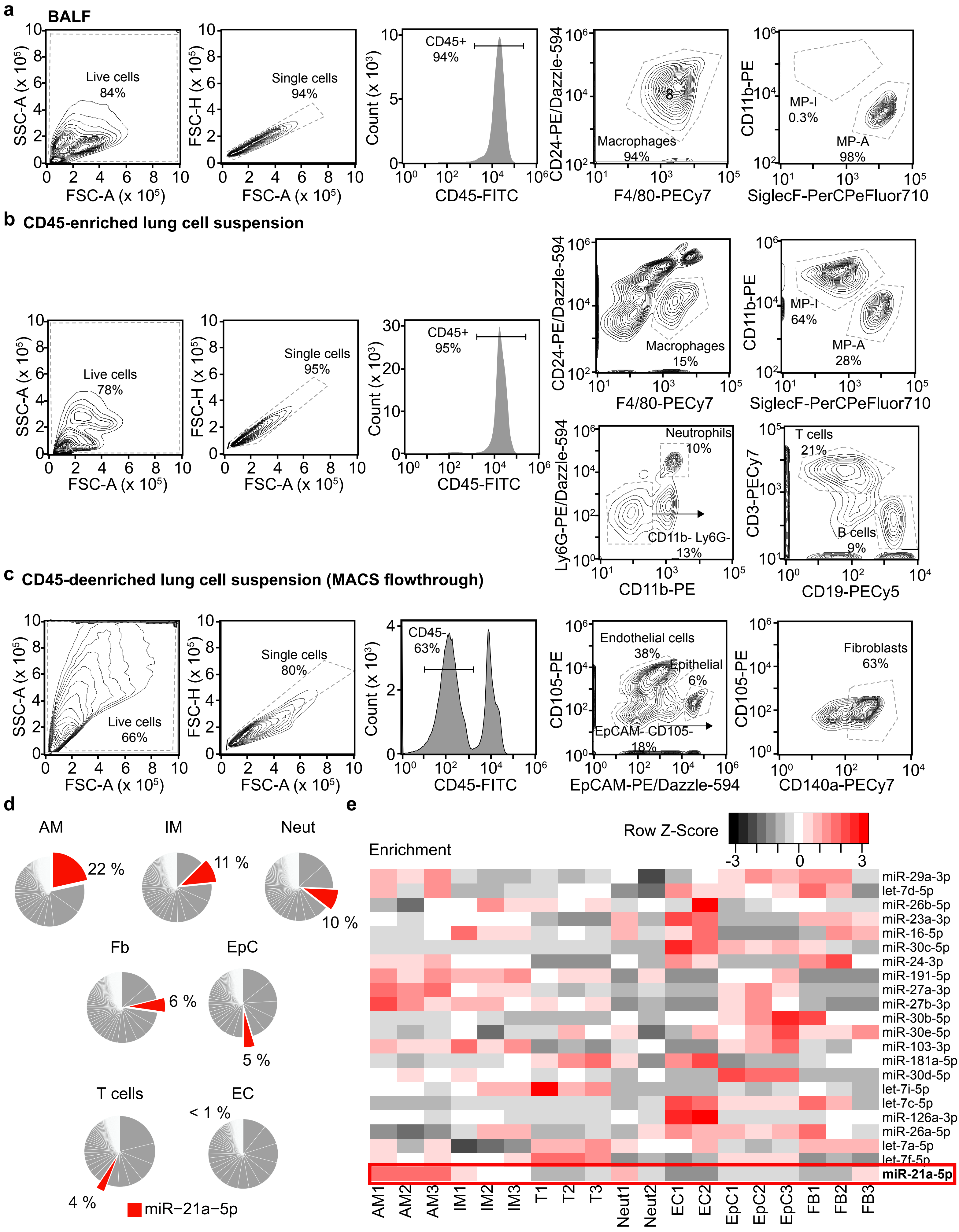

Fig. S2 | MiR-21 is highly abundant and enriched in pulmonary macrophages isolated from wild type mouse lungs. (a-c), Flow cytometry gating strategy for identifying **(a)** alveolar (MP-A) and interstitial (MP-I) macrophages in bronchoalveolar lavage fluid (BALF); **(b)** MP-A, MP-I, neutrophils, T cells and B cells in the CD45-enriched lung cell suspension and **(c)** endothelial cells, epithelial cells and fibroblasts in the CD45-deenriched lung cell suspensions. **d**, MicroRNA expression profiles determined by microRNA sequencing of different pulmonary cell types in mice. **e**, Heatmap of the top expressed miRs across different pulmonary cell types; n=2-3. Fb: fibroblasts, EC: endothelial cells, EpC: epithelial cells, Neut: neutrophils, IM: interstitial macrophages and AM: alveolar macrophages. Source data are provided as a Source Data file.

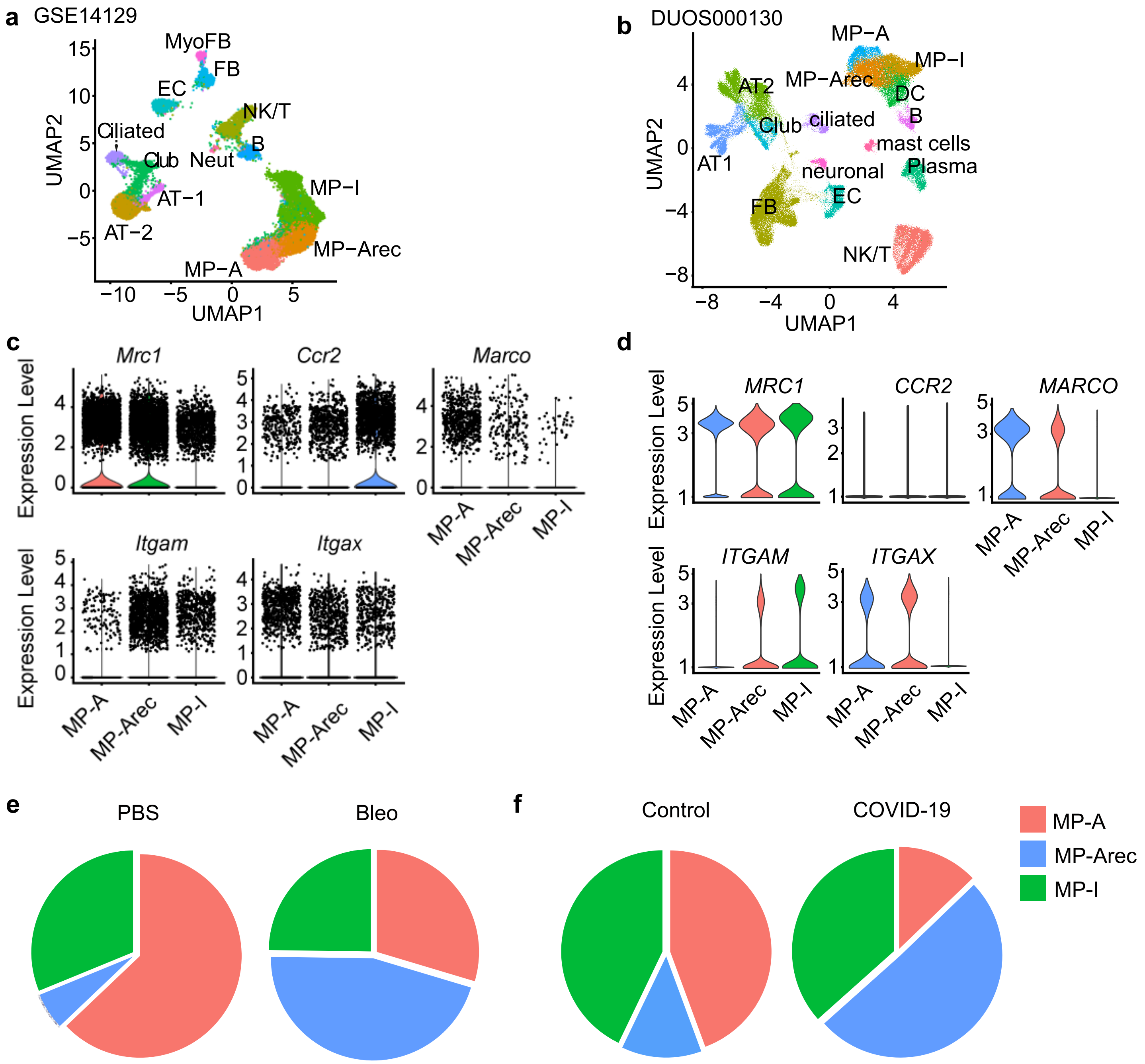

Fig. S3 | Comparative analysis of bleomycin and COVID-19 single cell datasets. a, Uniform manifold approximation and projection map (UMAP) plots for dimensionality reduction of the distribution of whole lung single cell transcriptomes after bleomycin injury (data from GSE141259). **b**, UMAP plots of COVID-19 lungs (data from DUOS000130). **c**, Violin plot showing the expression of marker genes in the MP-I, MP-A_rec and MP-A subclusters in bleomycin-induced lungs. **d**, Violin plot showing the expression of marker genes in the MP-I, MP-A_rec and MP-A subclusters in COVID-19 lungs. **e**, Frequency of MP-I, MP-A and MP-A_rec in control and bleomycin-treated lungs. **f**, Frequency of MP-I, MP-A and MP-A_rec in control and COVID-19 lungs. DC dendritic cells, DC-Itgae Itgae-positive dendritic cells, EC endothelial cells, EC-art endothelial cells-arteriole, EpC epithelial cells, FB fibroblasts, IgG immunoglobulin, MP-A alveolar macrophages, MP-I interstitial macrophages, Neut neutrophils, NK natural killer cells, pDC plasmacytoid dendritic cells and Peri pericytes.

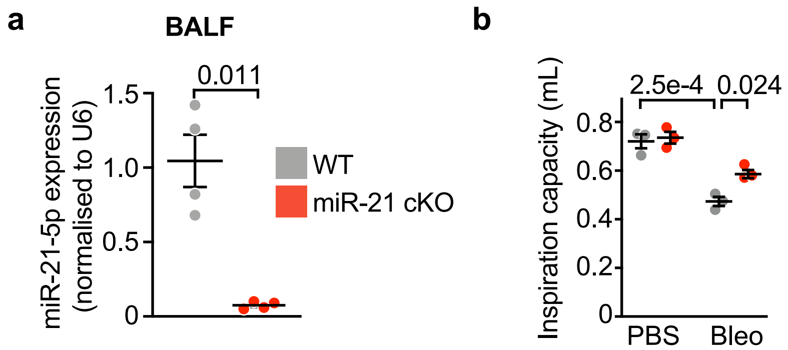

Fig. S4 | Characterization of mice with macrophage-specific deletion of miR-21. a, Relative expression of miR-21a-5p determined in bronchoalveolar lavage fluid (BALF) cells isolated from WT and miR-21 cKO mice; WT n=4 and miR-21 cKO n=4. **b**, Inspiration capacity in wild type and miR-21 cKO mice after bleomycin; WT PBS n=3, miR-21 cKO n=3, WT bleo n=3 and miR-21 cKO n=3. Data are mean ± SEM and individual values, and were analysed using (a) unpaired Student's t-test (two-tailed) with Welch's correction or (b) two-way ANOVA with Tukey's post test (two-sided). Source data are provided as a Source Data file.

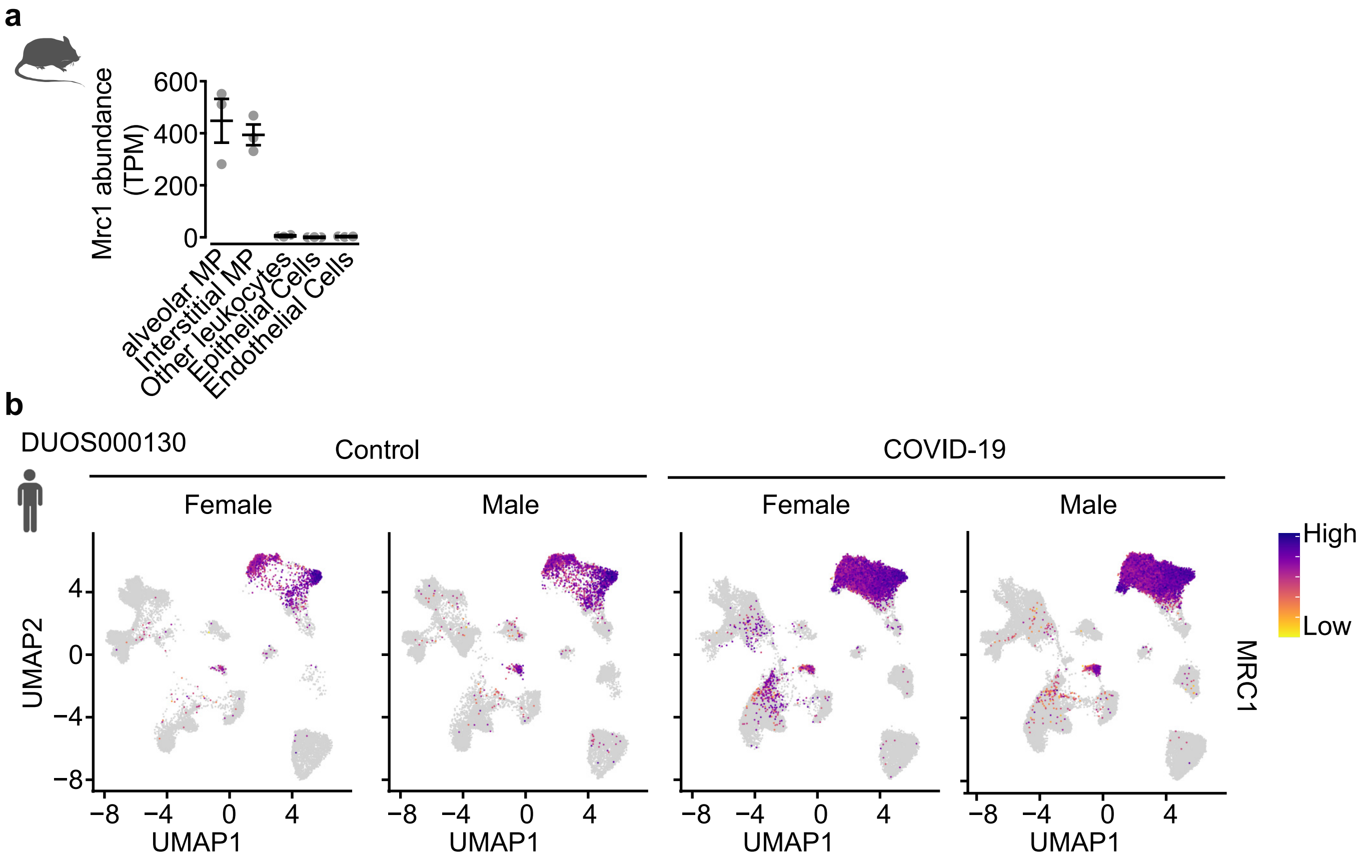

Fig. S5 | *Mrc1* is expressed in pulmonary macrophages in mice and humans. a, Relative expression of *Mrc1* in total reads per million (TPM) in lung cell types in mice; n=3 per cell type. Data are mean ± SEM and individual values. MP: macrophages. **b**, Feature plot showing the expression of *MRC1* in female and male individuals from human lungs (data: DUOS-000130) ; control n=7, COVID-19 n=19. Source data are provided as a Source Data file.

GSE14129

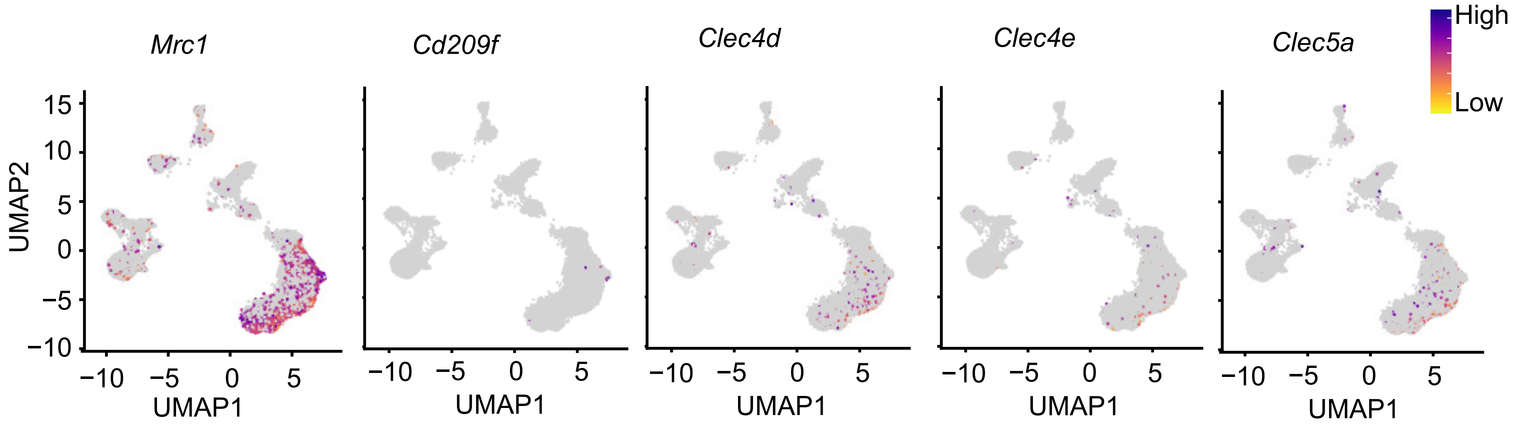

Fig. S6 | Feature plots showing expression of various C-type lectins (*Mrc1, Cd209f, Clec4d, Clec4e and Clec5a*) in mouse lungs after bleomycin-induced lung injury. Data from GSE14129.

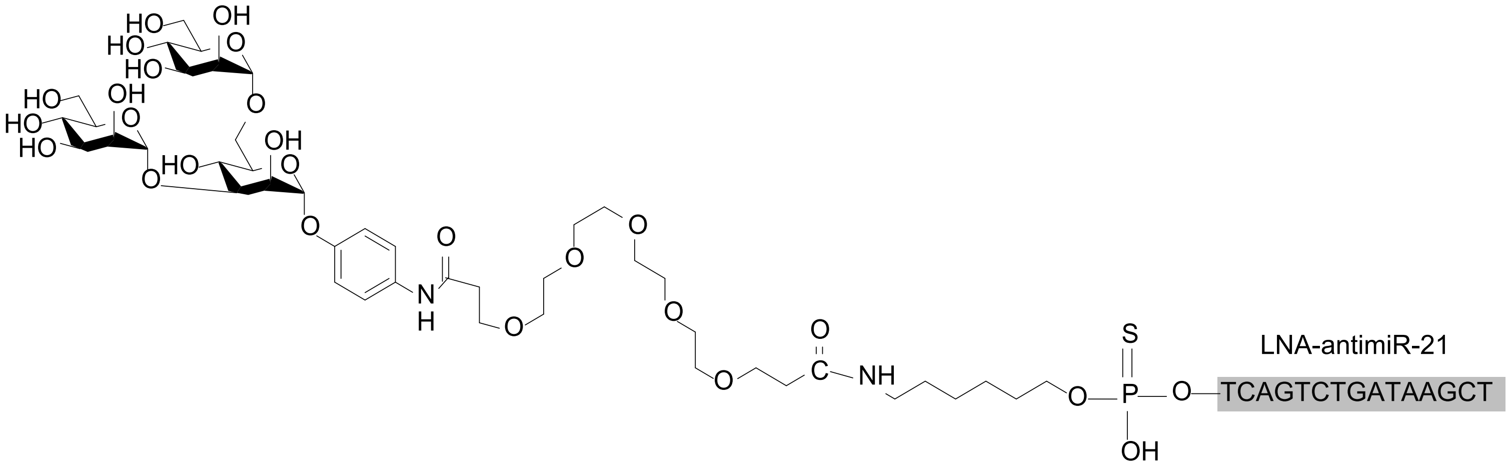

Fig. S7 | Chemical structure of trimannose-conjugated LNA-antimiR-21 (RCS-21) prepared using conventional chemistry.

Structure	Name	Binding score
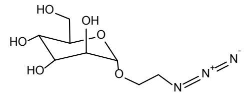	2-Azidoethyl α-D-mannopyranoside	-2.15
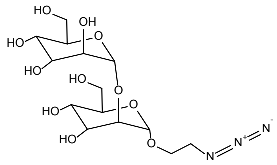	2-Azidoethyl 2-O-α-D-mannopyranosyl-D-mannopyranoside	-2.72
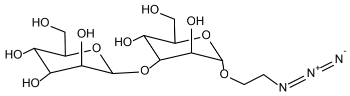	2-Azidoethyl 3-O-α-D-mannopyranosyl-D-mannopyranoside	-2.56
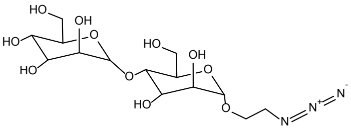	2-Azidoethyl 4-O-α-D-mannopyranosyl-D-mannopyranoside	-2.43
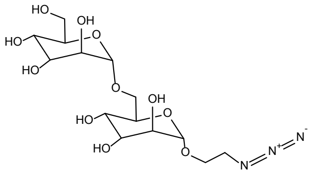	2-Azidoethyl 6-O-α-D-mannopyranosyl-D-mannopyranoside	-2.53
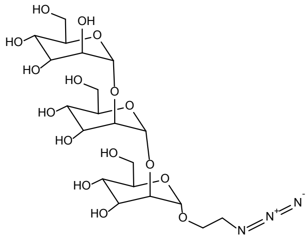	2-Azidoethyl 2-O-(2-O-α-D-mannopyranosyl)-α-D-mannopyranosyl-α-D-mannopyranoside	-2.82
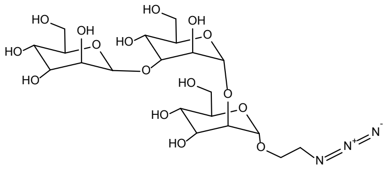	2-Azidoethyl 2-O-(3-O-α-D-mannopyranosyl)-α-D-mannopyranosyl-α-D-mannopyranoside	-3.12
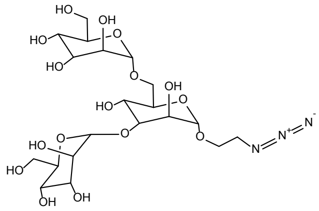	2-Azidoethyl 3,6-di-O-(α-D-mannopyranosyl)-α-D-mannopyranoside (branched trimannose RCS-21)	-3.22

Fig. S8 | Mannose saccharides included in *in silico* binding studies to CRD4 domain of MRC1.

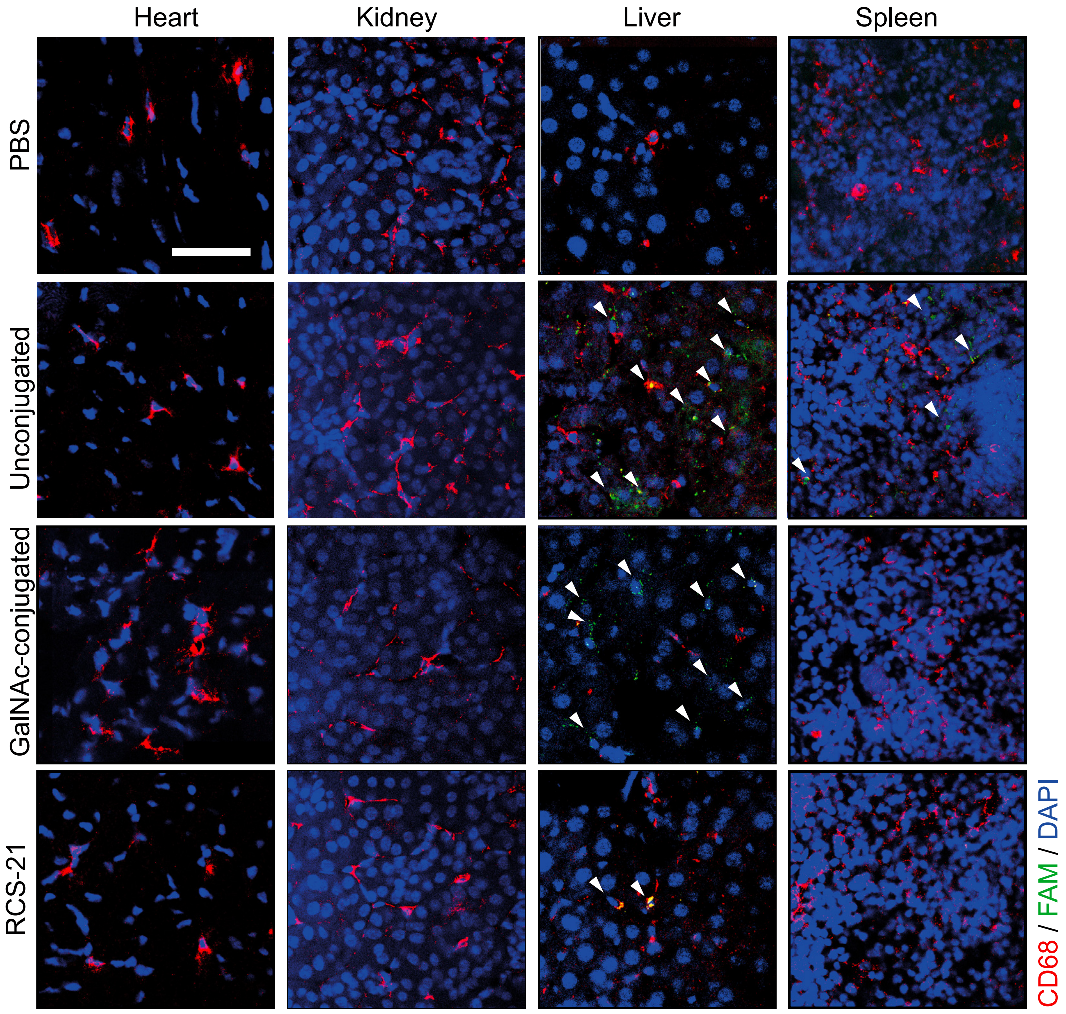

Fig. S9| Assessment of RCS-21-FAM in liver, kidney, heart and spleen. Representative immunofluorescent staining of 5 µm mouse tissue cryosections for CD68 as a marker for macrophages (PBS n=3, Unconjugated n=3, GalNAc-conjugated n=3 and RCS-21 n=3). Nuclei were stained with DAPI. Scale bar represents 50 µm. White arrows indicate FAM-positive macrophages. FAM, fluorescein amidites.

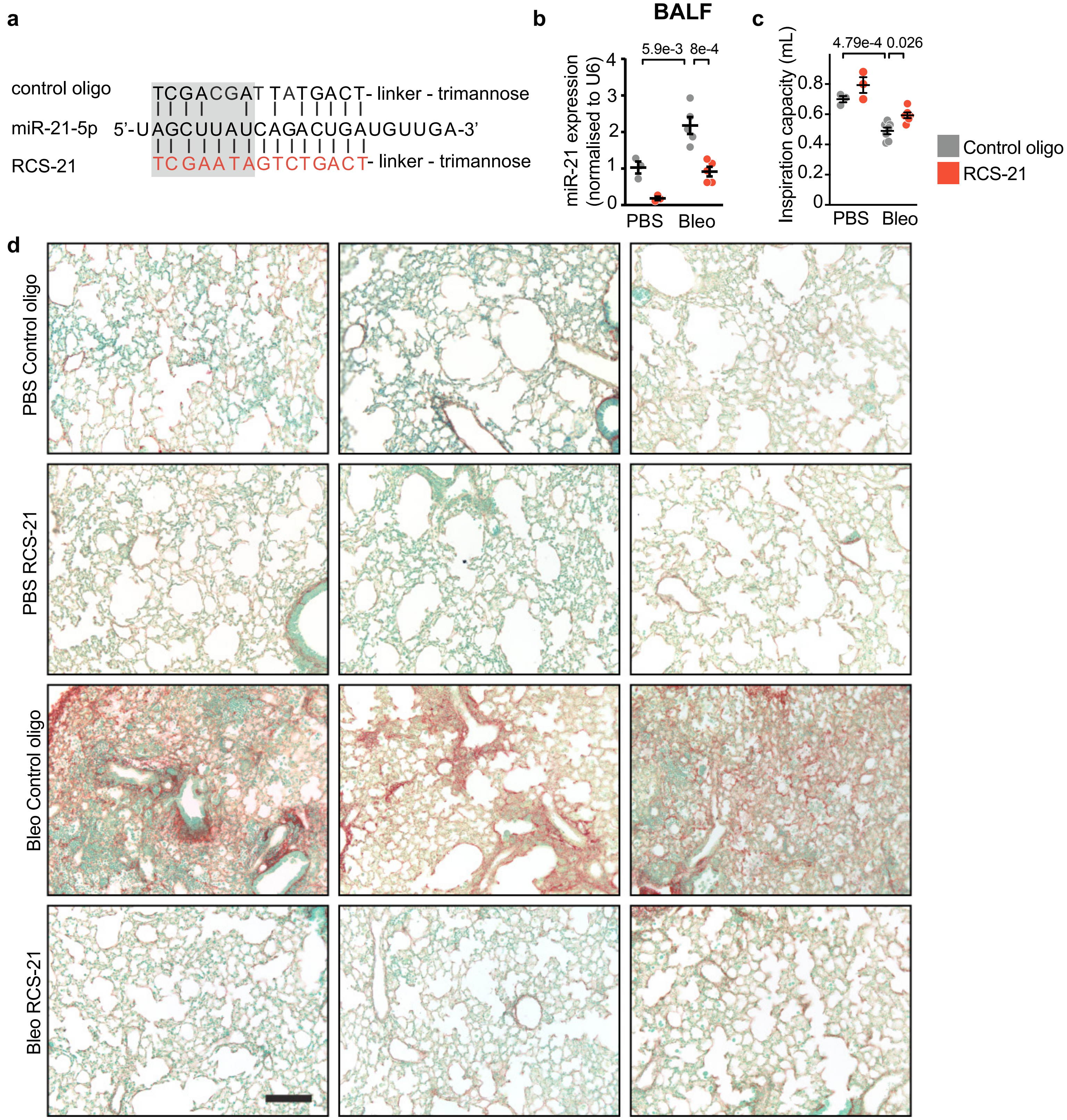

Fig. S10 | RCS-21 inhalation study. a, Sequences of miR-21, RCS-21 and control oligo used in therapeutic assay (as described in Fig. 4). **b**, Relative expression of miR-21a-5p determined in bronchoalveolar lavage fluid (BALF) cells isolated from RCS-21-treated and control-treated mice; PBS control oligo n=3, PBS RCS-21 n=3, Bleo control oligo n=5 and Bleo RCS-21 n=5. **c,** Lung function as indicated by inspiration capacity in mice treated with RCS-21 or control oligo 14 days after bleomycin. Control oligo: PBS n=3, bleo n=7. RCS-21: PBS n=3, bleo n=6. b-c, Data are mean ± SEM and individual values, and were analysed using two-way ANOVA with Tukey's post test (two-sided). Source data are provided as a Source Data file. **d,** Representative Fast green sirius red stainings of larger lung sections. Scale bar represents 100 µm. Control oligo: PBS n=3, bleo n=7. RCS-21: PBS n=3, bleo n=6.

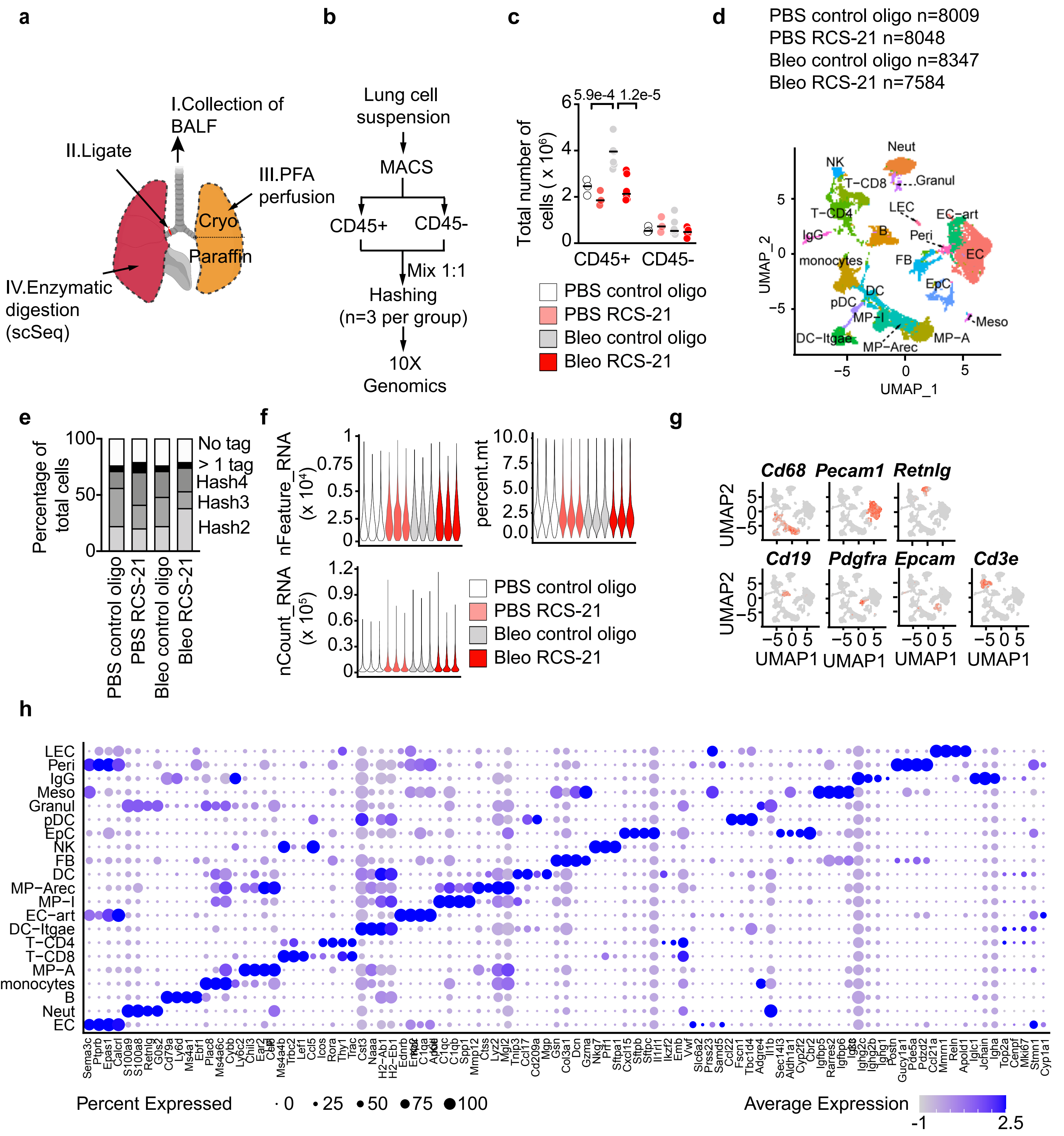

Fig. S11 | Single cell sequencing of RCS-21-treated mouse lungs after acute lung injury. **a**, Schematic illustration of the mouse lung regions used for different analysis. **b**, Experimental workflow of single cell RNA sequencing of pulmonary cells harvested from mice 14 days after bleomycin injury. Superior, middle and inferior lobes of the right lung were digested and were separated into leukocyte and non-leukocyte populations using CD45-based magnetic-activated cell sorter (MACS). Three mice from each group were selected for the single cell experiment. The two populations from each mouse were then mixed in the ratio of 1:1 and were multiplexed with TotalSeq A hashing antibodies. Cell suspensions from the three mice belonging to the same experimental group were pooled together and barcoded cDNA was then generated using 10X genomics Single Cell 3'-library kit, sequencing performed on NovaSeq and analysed using Cellranger and Seurat packages. **c**, Cell numbers of leukocyte (CD45-positive) and non-leukocytes (CD45-negative) fractions after MACS for the entire group. Control oligo: PBS n=3, bleomycin n=7. RCS-21: PBS n=3, bleomycin n=6. Data denote mean and individual values, and were analysed using 2-way ANOVA with Tukey's post test (two-sided). **d**, Uniform manifold approximation and projection map (UMAP) plots for dimensionality reduction of the distribution of single cell transcriptomes. Control oligo: PBS n=3, bleo n=3. RCS-21: PBS n=3, bleo n=3. DC dendritic cells, DC-Itgae Itgae-positive dendritic cells, EC endothelial cells, EC-art endothelial cells-arteriole, EpC epithelial cells, FB fibroblasts, IgG immunoglobulin, Meso mesothelial cells, LEC lymphatic endothelial cells, MP-A alveolar macrophages, MP-I interstitial macrophages, Neut neutrophils, NK natural killer cells, pDC plasmacytoid dendritic cells, Peri pericytes, T-CD4 CD4-positive T cells and T-CD8 CD8-positive T cells. **e**, Stacked bar graph showing proportion of cells after sample multiplexing. **f**, Violin plots showing number of genes, UMIs and mitochondrial content for all samples. **g**, Feature plot showing expression of known cell type markers denoting major cell populations: macrophages Cd68, endothelial cells Pecam1, neutrophils Retnlg, epithelial cells Epcam, T cells Cd3e, B cells Cd19, fibroblasts Pdgfra and natural killer cells Nkg7. **h**, Dotplot showing known cell type markers after sub-clustering. Source data are provided as a Source Data file.

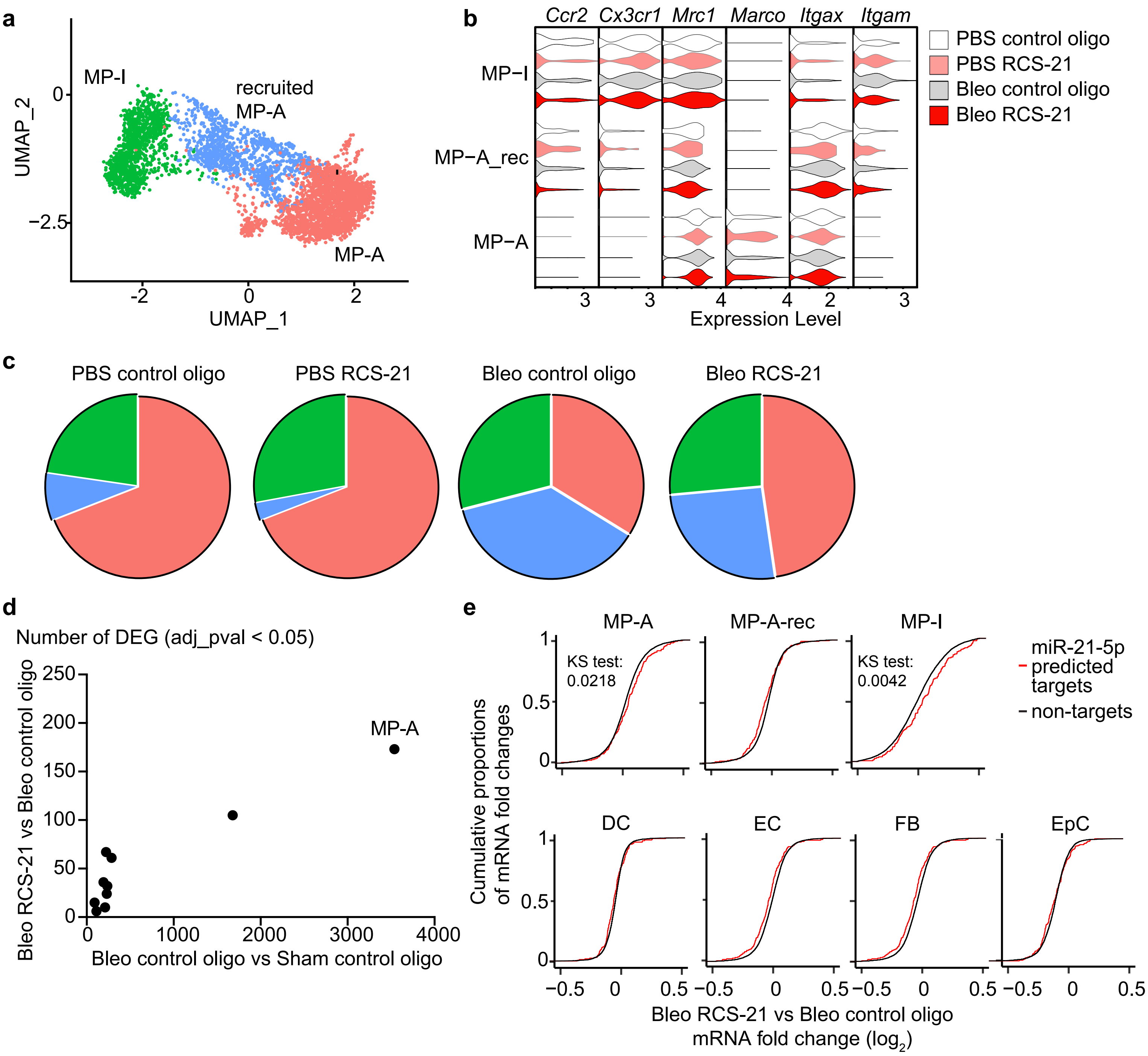

Fig. S12 | Further sub-clustering of the macrophage populations shows a decrease in recruited macrophages after RCS-21 treatment in bleomycin mice. **a**, Uniform manifold approximation and projection map (UMAP) plots for dimensionality reduction of the distribution of the macrophage single cell transcriptomes. Control oligo: PBS n=3, bleo n=3. RCS-21. **b**, Violin plot showing the expression of marker genes in the MP-I, MP-A_rec and MP-A subclusters. MP-A alveolar macrophages, MP-A_rec recruited macropahges, MP-I interstitial macrophages. **c**, Pie graphs showing the proportions of different macrophage clusters in different conditions. **d**, Frequency of significantly regulated differentially expressed genes across treatmets. cutoff: adj_pval < 0.05. Control oligo: PBS n=3, bleo n=3. RCS-21: PBS n=3, bleo n=3. **e**, Cumulative distribution curves represent miR-21-5p activity. Rightward shift of predicted miR-21 targets (red) indicates decreased miR-21 activity (RCS-21 vs. control). Statistical analysis was performed using a two-sided Kolmogorov-Smirnov (KS) test. DC dendritic cells, EC endothelial cells, EpC epithelial cells, FB fibroblasts, MP-A alveolar macrophages, MP-A-rec recruited macrophages, MP-I interstitial macrophages. Source data are provided as a Source Data file.

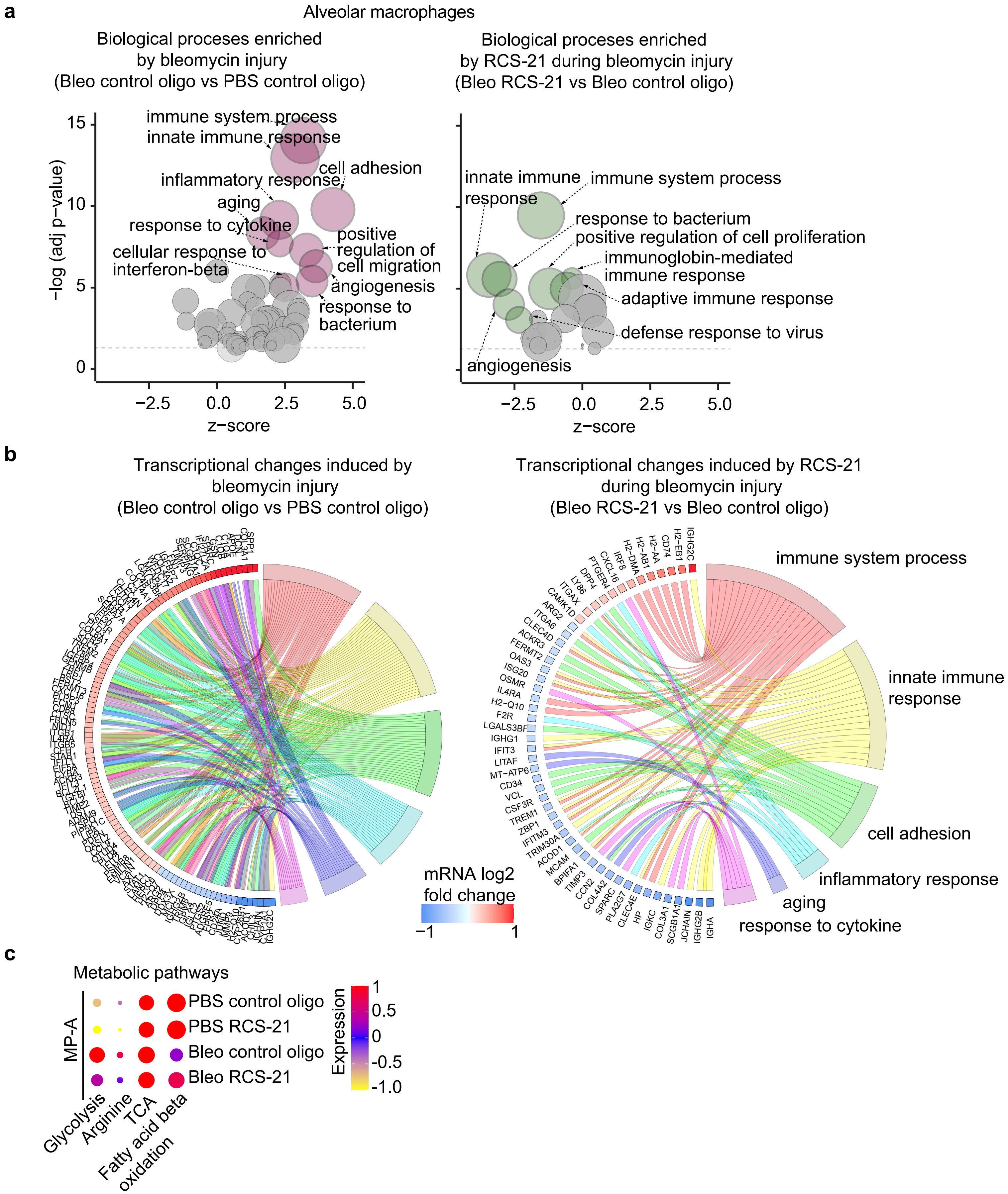

Fig. S13 | Gene ontology analysis of transcriptomes of alveolar macrophage cluster. a, Gene ontology (GO) enrichment analysis on biological processes of the top 200 deregulated genes in alveolar macrophages in Bleo control oligo vs PBS control oligo and Bleo RCS-21 vs Bleo control oligo. Gray dotted line indicates adjusted P-value <0.05. DAVID uses a modified Fisher's exact test for statistics. **b**, Chord diagram showing the deregulated genes and their associated GO terms. **c**, Dotplot showing the module scores for signature gene set for metabolic pathways in macrophage clusters. Source data are provided as a Source Data file.

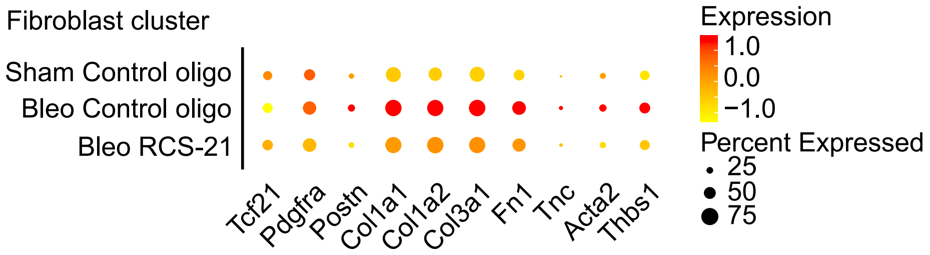

Fig. S14 | Expression of extracellular matrix-related genes in fibroblast cluster. Dot plot showing expression of fibroblast-related genes indicating fibroblast activation after bleomycin and repression of this signalling after treatment with RCS-21.

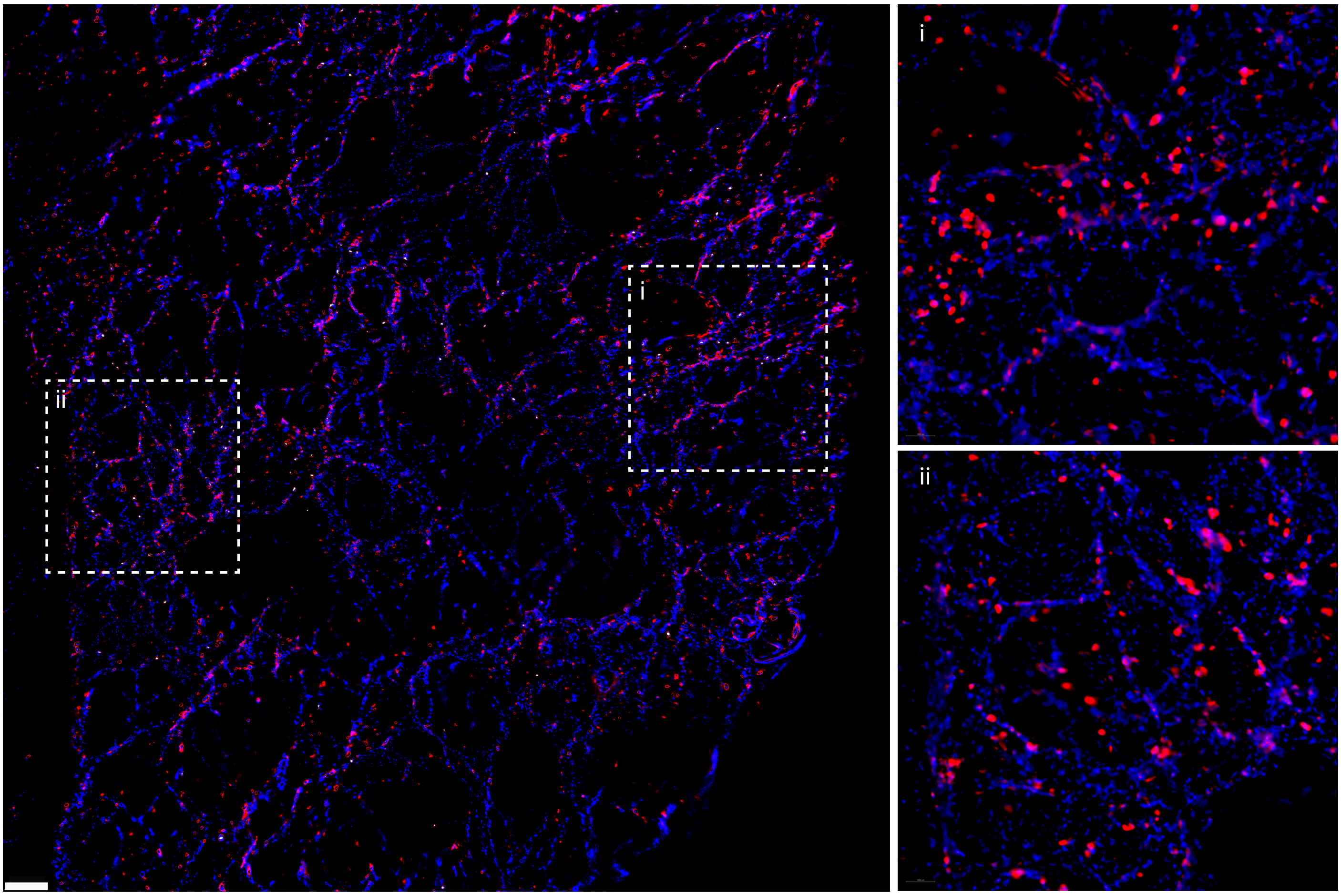

Fig. S15 | Representative macrophage staining in hPCLS. hPCLS were stained with DAPI (nucleus) and Cd68 (macrophage marker); n=2. Scalebar represents 200µm.

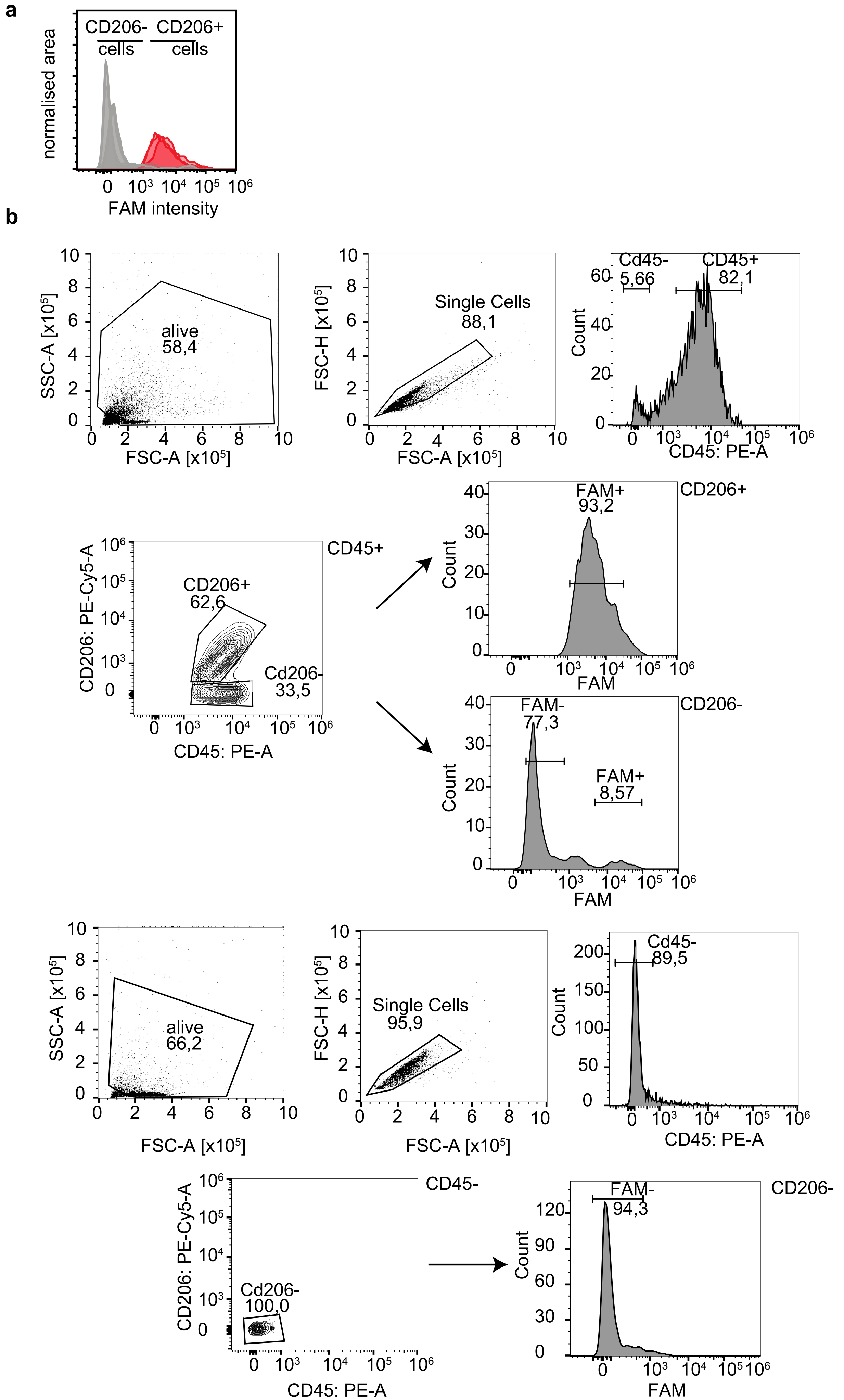

Fig. S16 | hPCLS were treated with FAM-labelled RCS-21. a, hPCLS were treated with FAM-labelled RCS-21 and 2hrs later FAM signal was assessed in CD206+ and CD206- cells; n=3 replicates with 8 hPCLS for each replicate. **b**, Flow cytometry gating strategy to asess FAM signal in CD206+ cells and CD206- cells in the CD45+ fraction. **c**, Flow cytometry gating strategy to asess FAM signal in CD206+ cells and CD206- cells in the CD45- fraction.

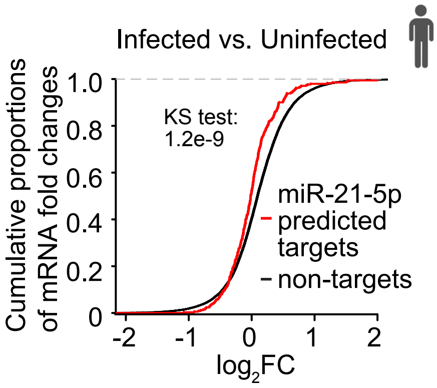

Fig. S17 | Increased miR-21 activity in human precision cut lung slices (hPCLS) 24 hours after infection with SARS-CoV-2 (Omicron). Cumulative-distribution curves. Leftward shift of miR-21 targets (red) indicates increased miR-21 activity (Infected vs Uninfected). Statistical analysis was performed using two-sided Kolmogorov-Smirnov (KS) test; n(uninfected)=5, n(infected)=6. Source data are provided as a Source Data file.

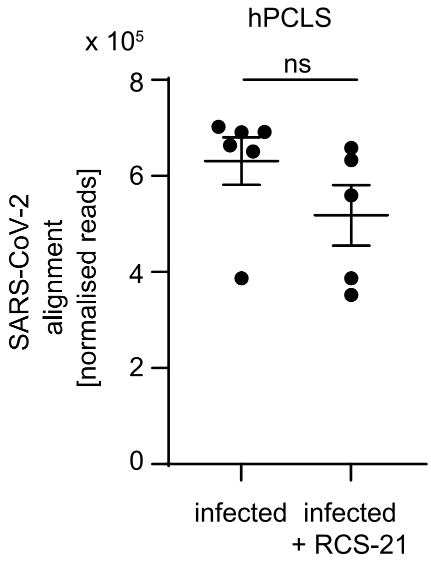

Fig. S18 | Viral load in SARS-CoV-2 infected and infected + RCS-21 slices. RNA-seq data were aligned to SARS-CoV-2 reference genome and normalised; n(infected)=6, n(infected+RCS-21)=5. Data are mean ± SEM and individual values and were analysed using Student's t-test (two-tailed). Source data are provided as a Source Data file.

Sample ID	Patient ID	hours after post mortem collection	Status	SARS-CoV-2 aligned reads* (TPM)
C1	2827/20	0-48h pm	non-COVID-19 controls	1
C2	2822/20	0-48h pm	non-COVID-19 controls	0
C3	2820/20	0-48h pm	non-COVID-19 controls	0
C5	2816/20	0-48h pm	non-COVID-19 controls	3
C6	391/21	0-48h pm	non-COVID-19 controls	0
C7	364/21	0-48h pm	non-COVID-19 controls	0
C10	355/21	0-48h pm	non-COVID-19 controls	0
C13	356/21	0-48h pm	non-COVID-19 controls	0
C14	360/21	0-48h pm	non-COVID-19 controls	0
C1_H	S2063-20	0-48h pm	non-COVID-19 controls	1
C2_H	S0609-20	0-48h pm	non-COVID-19 controls	240
C4_H	S2067-20	0-48h pm	non-COVID-19 controls	36
L6	2516/20	0-48h pm	COVID-19	33156
L8	2749/20	0-48h pm	COVID-19	340103
L9	2760/21	0-48h pm	COVID-19	333430
L13	2796/20	0-48h pm	COVID-19	583564
L14	2687/20	0-48h pm	COVID-19	658284
L15	2748/20	0-48h pm	COVID-19	99241
L16	2420/20	0-48h pm	COVID-19	5875
L22	2192/20	0-48h pm	COVID-19	794921
L23	2392/20	0-48h pm	COVID-19	12227
L24	2671/20	0-48h pm	COVID-19	836089
L25	2727/20	0-48h pm	COVID-19	452047
M3	S0568-20	0-48h pm	COVID-19	678254
M4	S2060-20	0-48h pm	COVID-19	441388
M5	S0587-20	0-48h pm	COVID-19	57072

Supplementary Table 1: Donor metadata (post mortem study).
* Transcripts per millions < 1000 were considered as negative. Average age of the control group was 72 and for the COVID-19 group 76. Both groups include females and males.

Sample ID	Patient ID	Collection	Status
hPCLSCB170122	M170122	during surgery	lung tissue from patients who underwent thoracic surgery
hPCLSCB180122	A180122	during surgery	lung tissue from patients who underwent thoracic surgery
hPCLSCB040722	M040722	during surgery	lung tissue from patients who underwent thoracic surgery
hPCLSCB050722	A050722	during surgery	lung tissue from patients who underwent thoracic surgery
hPCLSCB280323	M280323	during surgery	lung tissue from patients who underwent thoracic surgery

Supplementary Table 2: Donor metadata (hPCLS study).
Average age of the patient cohort was 72 and the cohort consisted of females and males.
