# Supplementary material for: Trimannose-coupled antimiR-21 for macrophage-targeted inhalation treatment of acute inflammatory lung damage
Source: Nat Commun. 2023 Jul 28;14:4564. doi: 10.1038/s41467-023-40185-1 (PMC10382532; doi:10.1038/s41467-023-40185-1)
Supplement: Supplementary file 3 — Reporting Summary [file 41467_2023_40185_MOESM3_ESM.pdf]

nature portfolio

Corresponding author(s): Stefan Engelhardt

Last updated by author(s): July 7, 2023

Reporting Summary

Nature Portfolio wishes to improve the reproducibility of the work that we publish. This form provides structure for consistency and transparency in reporting. For further information on Nature Portfolio policies, see our Editorial Policies and the Editorial Policy Checklist.

Statistics

For all statistical analyses, confirm that the following items are present in the figure legend, table legend, main text, or Methods section.

n/a	Confirmed	
☐	☒	The exact sample size (*n*) for each experimental group/condition, given as a discrete number and unit of measurement
☐	☒	A statement on whether measurements were taken from distinct samples or whether the same sample was measured repeatedly
☐	☒	The statistical test(s) used AND whether they are one- or two-sided<br>*Only common tests should be described solely by name; describe more complex techniques in the Methods section.*
☐	☒	A description of all covariates tested
☐	☒	A description of any assumptions or corrections, such as tests of normality and adjustment for multiple comparisons
☐	☒	A full description of the statistical parameters including central tendency (e.g. means) or other basic estimates (e.g. regression coefficient) AND variation (e.g. standard deviation) or associated estimates of uncertainty (e.g. confidence intervals)
☐	☒	For null hypothesis testing, the test statistic (e.g. *F*, *t*, *r*) with confidence intervals, effect sizes, degrees of freedom and *P* value noted<br>*Give P values as exact values whenever suitable.*
☒	☐	For Bayesian analysis, information on the choice of priors and Markov chain Monte Carlo settings
☒	☐	For hierarchical and complex designs, identification of the appropriate level for tests and full reporting of outcomes
☒	☐	Estimates of effect sizes (e.g. Cohen's *d*, Pearson's *r*), indicating how they were calculated

Our web collection on statistics for biologists contains articles on many of the points above.

Software and code

Policy information about availability of computer code

Data collection	Flow cytometric data was collected in Sony SH800 instrument using Sony software. Lung function measurements was assessed on flexiVent device that is operated by flexiWare v8.1 software.
Data analysis	RNA-Seq analysis:<br>Bam filter v 0.5.9<br>CellRanger v 6.0.0<br>Cutadapt v4.0+galaxy0<br>DeSeq2 v 1.32.0<br>edgeR v 3.32.1<br>fastp v 23.2<br>Flexbar v 3.5.0<br>Galaxy platform v 22.05<br>ggplot2 v 3.3.6<br>GOplot v 1.0.2<br>HISAT2 v 2.1.0<br>MiRDeep2 v 2.0.0<br>R v 4.0.1<br>RUVSeq v 1.26.0<br>Seurat v 4.1.0

STAR v 2.7.8a
StringTie v 2.1.1
StringTie merge v 2.1.1
TrimGalore! v 0.6.7
violplot v 0.3.7

Databases used:
miRbase v 22.1
TargetScanHuman 7.2
TargetScanMouse 7.2
DAVID bioinformatics tool v 6.8

Image analysis:
FIJI ImageJ v 1.53n
MetaMorph v 7.10.1.161

Other:
ChemDraw v 20.0
flexiWare v 8.1
FlowJo v 10.7.1
Prism v 8.4.2/v 9.5.3
ProteinsPlus platform

For manuscripts utilizing custom algorithms or software that are central to the research but not yet described in published literature, software must be made available to editors and reviewers. We strongly encourage code deposition in a community repository (e.g. GitHub). See the Nature Portfolio guidelines for submitting code & software for further information.

Data

Policy information about availability of data

All manuscripts must include a data availability statement. This statement should provide the following information, where applicable:
- Accession codes, unique identifiers, or web links for publicly available datasets
- A description of any restrictions on data availability
- For clinical datasets or third party data, please ensure that the statement adheres to our policy

All raw and processed NGS data are available in GEO under the accession number GSE235136. All source data is provided with this paper.

Human research participants

Policy information about studies involving human research participants and Sex and Gender in Research.

Reporting on sex and gender	Post mortem samples: we used males and females in our study<br>for slices: we used males and females in our study
Population characteristics	See Data Table S1 and S2 in the supplementary information file.
Recruitment	Post mortem samples were recruited from the Institutes of Rechtsmedizin at UKE and LMU and no self-selection was involved.<br><br>Lung tissue for preparing human lung slices were recruited from patients who underwent surgery at the Thoracic Surgery, Klinikum rechts der Isar, Technische Universität München and have signed the written informed consent. No self-selection was involved. We recruited as many patients as possible. Harvesting of samples was not influenced by sex, age, co-morbidities or pre-treatments.
Ethics oversight	For all the experiments, an ethic committee approval (approval number 59/21S, Ethikkommission der Fakultät für Medizin) from the Technical University of Munich and a signed written informed consent were obtained.

Note that full information on the approval of the study protocol must also be provided in the manuscript.

Field-specific reporting

Please select the one below that is the best fit for your research. If you are not sure, read the appropriate sections before making your selection.

[x] Life sciences [ ] Behavioural & social sciences [ ] Ecological, evolutionary & environmental sciences

For a reference copy of the document with all sections, see nature.com/documents/nr-reporting-summary-flat.pdf

Life sciences study design

All studies must disclose on these points even when the disclosure is negative.

Sample size	Post mortem samples:<br>human lung tissue (COVID-19) (n=14, Donors= L6,L8-9,L13-16,L22_25, M3-M5)<br>human lung tissue (controls) (n=12, Donors= C1-3,C5-7,C10, C13-14,C1_H-C2_H,C4_H)<br><br>As these are post mortem samples from COVID-19 donors and control donors we collected as many samples as possible during our experimental period.<br><br>For preparation of slices:<br>Donors (n=5, Donors= M170122, A180122, M040722, A050722, M280323)<br>An exact sample size calculation by PowerAnalysis for RNA-seq data is still challenging. By increasing the sequencing depth and using paired-end reads we increased the statistical power of our findings. In spite of the fact that a larger sample size would provide even more statistical power, the scarcity of human lung tissue samples precluded us from doing so.<br><br>Mice studies after treatment with RCS-21: Sample sizes were determined by a statistical institute based on previous results obtained by our collaboration group. With a standard deviation of 20% and effect size of 40% in a previous study gave us a statistical power of 0.82 (up to 8 animals per experimental group).
Data exclusions	Post mortem samples:<br>Few samples with poor RNA quality were not used for sequencing analysis because degraded RNA can produce false results. This study excluded lung tissues harvested from COVID-19 patients with low viral reads (total reads per million, TPM <1000 for SARS-CoV2).<br><br>Experiments on human precision cut slices infected with SARS-CoV2 and treated with RCS-21:<br>Few samples with poor RNA quality were not used for sequencing analysis because degraded RNA can produce false results.<br><br>Mice studies:<br>Few samples from sorted cells with poor RNA quality were not used for sequencing analysis because degraded RNA can produce false results.<br>No experimental mice treated with RCS-21 and bleomycin were excluded from the study. For the single-cell RNAseq data, however, cells were filtered for quality control.
Replication	Post mortem samples:<br>We could not replicate samples, since these are post mortem samples from human COVID-19 donors.<br><br>Human precision cut lung slices: All experiments were repeated at least four times using hPCLS generated from at least two different donors per experimental condition.<br><br>Mice study: each experimental group contained more than 3 biological replicates.<br><br>All attempts at replication were successful.
Randomization	Post mortem samples: Since no subgroups within the Control and the COVID-19 group were created, randomization was not possible.<br><br>For slices: Allocation of hPCLS to the various experimental groups were completely randomized.<br><br>Animals were randomly assigned to the experimental groups at the time of bleomycin/PBS application for lung injury and for the application of antimiR using flexiVent device. After the randomization exercise, PBS applications were carried out prior to bleomycin applications using the microsprayer to induce lung injury or while applying the antimiR using the flexiVent nebulizer unit to facilitate washing the units thoroughly and prevent cross-contamination of reagents between experimental groups.<br><br>For scRNA-seq experiments, no randomization was performed in choosing three mice per experimental group to mitigate the potential risk of multiplexing failures resulting in compromised data quality or loss of the entire sequencing dataset.<br>To minimize covariates, the single-cell experiment on mouse lung tissue after RCS-21 application was carefully designed and conducted in one batch. All critical steps, including organ harvesting, cell isolation and processing, and the first step of the 10X scSeq protocol (Gel-bead-in-emulsion (GEMs) preparation), were performed on the same day. In addition, the libraries were sequenced on a single Novaseq flow cell. Individual datasets did not demonstrate discernible batch effects, as evidenced by consistent outcomes across multiple parameters. Both the multiplexing ratio, representing the proportion of cells from different mouse samples within each 10X single-cell reaction, and the leukocyte:non-leukocyte ratio remained consistent across all samples. Clustering analysis revealed comparable cell type identification and clustering patterns across samples, confirming the absence of batch effects.
Blinding	For post mortem tissue: Analysis was not blinded. All stainings, histological assessment and scoring were blinded.<br><br>For slices: Experiments and data analysis were not blinded. All stainings, histological assessment and scoring were blinded.<br><br>For mice experiments, person carrying out the lung function was blinded for the antimiR applied to the mice. All stainings, histological assessment and scoring were blinded.<br>No blinding was performed for scRNA-seq experiments for mice cell suspensions used in library generation, processing and data analysis.

Three mice per experimental group for single cell sequencing were chosen depending on cell viability (> 85%) for both leukocyte and non-leukocyte fractions obtained from this mice.

Sequencing analysis is typically not performed in a blinded manner because of several practical and methodological reasons (including full understanding of the datasets, providing of metadata).

Reporting for specific materials, systems and methods

We require information from authors about some types of materials, experimental systems and methods used in many studies. Here, indicate whether each material, system or method listed is relevant to your study. If you are not sure if a list item applies to your research, read the appropriate section before selecting a response.

Materials & experimental systems

n/a	Involved in the study	
☐	☒	Antibodies
☒	☐	Eukaryotic cell lines
☒	☐	Palaeontology and archaeology
☐	☒	Animals and other organisms
☒	☐	Clinical data
☒	☐	Dual use research of concern

Methods

n/a	Involved in the study	
☒	☐	ChIP-seq
☐	☒	Flow cytometry
☒	☐	MRI-based neuroimaging

Antibodies

Antibodies used

Antibodies for flow cytometry:
Anti-CD3-PECy7 (clone 17A2, diluted 1:100, Cat #100220, BioLegend, SanDiego, USA),
Anti-CD11b-PE (clone M1/70; diluted 1:160, Cat #101208, BioLegend, SanDiego, USA),
Anti-CD19-PECy5 (clone 1D3, diluted 1:80, Cat # 15-0193-82, Thermofisher Scientific, Darmstadt, Germany),
Anti-CD24-PE/Dazzle 594 (clone M1/69; diluted 1:80, Cat #101838 BioLegend, SanDiego, USA),
Anti-CD45-FITC (clone 30-F11, Cat #103108, diluted 1:100, BioLegend, SanDiego, USA),
Anti-CD45-PE (clone HI30, diluted 1:20, Cat #304008, BioLegend, SanDiego, USA),
Anti-CD45-PECy5 (clone 30-F11, diluted 1:80, Cat #103110, BioLegend, SanDiego, USA),
Anti-CD105-PE (clone MJ7/18, diluted 1:100, Cat #12-1051-82 ,Thermofisher Scientific, Darmstadt, Germany),
Anti-CD140a-PECy7 (clone APA5, diluted 1:100, Cat #25-1401-82, Thermofisher Scientific, Darmstadt, Germany),
Anti-CD206-PE-Cy5 (clone 15-2, diluted 1:20, Cat #2205540, Sony Biotechnology, San Jose, USA),
Anti-CD206-PE/Dazzle 594 (clone C068C2, diluted 1:40, Cat #141732 , BioLegend, SanDiego, USA),
Anti-E-cadherin-PerCP/Cy5.5 (clone DECMA-1, diluted 1:20, Cat #147318 , BioLegend, SanDiego, USA),
Anti-EpCAM-PE/Dazzle 594 (clone G8.8, diluted 1:100, Cat #118236, BioLegend, SanDiego, USA),
Anti-F4/80-PECy7 (clone BM8, diluted 1:100, Cat #25-4801-82, Thermofisher Scientific, Darmstadt, Germany),
Anti-Ly6G-PE/Dazzle 594 (clone (1A8, diluted 1:80, Cat #127648, BioLegend, SanDiego, USA),
Anti-SiglecF-PerCP-eFluor-710 (clone 1RNM44N, diluted 1:100, Cat #46-1702-82, Thermofisher Scientific, Darmstadt, Germany)

Antibodies for Immunoflourescence:
Anti-CD68 (FA-11, diluted 1:50 in 3% goat serum/PBS, Cat #MCA1957,BIO-RAD, Feldkirchen, Germany),
Anti-MRC1 (polyclonal, diluted 1:100 in 3% goat serum/PBS,Cat #ab64693 ,abcam, Cambridge, UK),
Invitrogen™ CD68 Monoclonal Ab (KP1, diluted 1:100, Cat #14-0688-82,Thermofisher Scientific, Darmstadt, Germany),
Invitrogen™ Goat anti-mouse IgG (H+L), AlexaFluor® 488 (diluted 1:100 in 0.1% Tween20/PBS, Cat #A-11029, Thermofisher Scientific, Darmstadt, Germany),
Invitrogen™ Goat anti-rabbit IgG (H+L), AlexaFluor® 647 (diluted 1:100 in 0.1% Tween20/PBS, Cat #A-2124 ,Thermofisher Scientific, Darmstadt, Germany),
Invitrogen™ Goat anti-rat IgG (H+L), AlexaFluor® 647 (diluted 1:100 in 0.1% Tween20/PBS, Cat #A-21247, Thermofisher Scientific, Darmstadt, Germany)
Antibodies for RNA single-cell sequencing:
TotalSeq™-A0302 anti-mouse Hashtag 2 (H2) Antibody, Cat #155803 , BioLegend, SanDiego, USA
TotalSeq™-A0303 anti-mouse Hashtag 3 (H3)Antibody, Cat #155805, BioLegend, SanDiego, USA
TotalSeq™-A0304 anti-mouse Hashtag 4 (H4) Antibody , Cat #155807 BioLegend, SanDiego, USA

Statements from the websites for the according antibodies:

For flow cytometry:
a) BioLegend: Each lot of these antibodies is quality control tested by immunofluorescent staining with flow cytometric analysis. For flow cytometric staining, the suggested use of these reagents is ≤0.06-1 µg per million cells in 100 µl volume. It is recommended that the reagent be titrated for optimal performance for each application.

b)Thermofisher Scientific.

Applications Reported: This eBio1D3 (1D3) antibody has been reported for use in flow cytometric analysis. Applications Tested: This eBio1D3 (1D3) antibody has been tested by flow cytometric analysis of mouse splenocytes. This can be used at less than or equal to 0.25 µg per test. A test is defined as the amount (µg) of antibody that will stain a cell sample in a final volume of 100 µL. Cell number should be determined empirically but can range from 10^5 to 10^8 cells/test. It is recommended that the antibody be carefully titrated for optimal performance in the assay of interest.

Applications Reported: This MJ7/18 antibody has been reported for use in flow cytometric analysis.
Applications Tested: This MJ7/18 antibody has been tested by flow cytometric analysis of mouse brain-derived endothelial (bEnd. 3) cells and splenocytes. This can be used at less than or equal to 0.5 µg per test. A test is defined as the amount (µg) of antibody that will stain a cell sample in a final volume of 100 µL. Cell number should be determined empirically but can range from 10^5 to 10^8 cells/test. It is recommended that the antibody be carefully titrated for optimal performance in the assay of interest.

Applications Reported: This APA5 antibody has been reported for use in flow cytometric analysis.
Applications Tested: This APA5 antibody has been tested by flow cytometric analysis of NIH/3T3 cells. This can be used at less than or equal to 0.125 µg per test. A test is defined as the amount (µg) of antibody that will stain a cell sample in a final volume of 100 µL. Cell number should be determined empirically but can range from 10^5 to 10^8 cells/test. It is recommended that the antibody be carefully titrated for optimal performance in the assay of interest.

Applications Reported: This BM8 antibody has been reported for use in flow cytometric analysis.
Applications Tested: This BM8 antibody has been tested by flow cytometric analysis of mouse resident peritoneal exudate cells. This can be used at less than or equal to 0.5 µg per test. A test is defined as the amount (µg) of antibody that will stain a cell sample in a final volume of 100 µL. Cell number should be determined empirically but can range from 10^5 to 10^8 cells/test. It is recommended that the antibody be carefully titrated for optimal performance in the assay of interest.

Applications Reported: This 1RNM44N antibody has been reported for use in flow cytometric analysis.
Applications Tested: This 1RNM44N antibody has been tested by flow cytometric analysis of mouse thioglycolate-elicited peritoneal exudate cells. This can be used at less than or equal to 0.125 µg per test. A test is defined as the amount (µg) of antibody that will stain a cell sample in a final volume of 100 µL. Cell number should be determined empirically but can range from 10^5 to 10^8 cells/test. It is recommended that the antibody be carefully titrated for optimal performance in the assay of interest.

Sony Biotechnology:
Applications: Flow Cytometry
Recommended Usage:
Each lot of this antibody is quality control tested by immunofluorescent staining with flow cytometric analysis. Test size products are transitioning from 20 microL to 5 microL per test. Please check your vial or your CoA to find the suggested use of this reagent per million cells in 100 microL staining volume or per 100 microL of whole blood. It is recommended that the reagent be titrated for optimal performance for each application.

For IF:

BIO-RAD
This product has been reported to work in the following applications (including IF). This information is derived from testing within our laboratories, peer-reviewed publications or personal communications from the originators. Please refer to references indicated for further information. For general protocol recommendations, please visit the antibody protocols page.

abcam:
Our Abpromise guarantee covers the use of ab64693 in the following tested applications (including IF). Use a concentration of 1 µg/ml. Permeabilise with Tween (0.1%).

ThermoFisher Scientific:
CD68
Applications Reported: This KP1 antibody has been reported for use in flow cytometric analysis, immunoprecipitation, western blotting, immunohistochemical staining of frozen tissue sections, and immunohistochemical staining of formalin-fixed paraffin embedded tissue sections.

Validation

The antibodies used for this study were purchased from companies. All antibodies were validated by the manufacturers and/or cited in publications (provided on the manufacturer's websites). On manufacture's recommendation we titrated the antibodies according to our experimental setup.

For TotalSeqA studies used for multiplexing samples in scSeq experiment, antibodies (IgG) were tested using fluorescent conjugates to determine dilutions that led to staining slightly above background.

Animals and other research organisms

Policy information about studies involving animals; ARRIVE guidelines recommended for reporting animal research, and Sex and Gender in Research

Laboratory animals

10-12 weeks old wild type female mice on C57BL/6N background were used in all animal experiments. Mice were housed with a 12h light/dark cycle at 40-60% humidity. Animals were allowed food (altromin, 1328) and water ad libitum.

	Macrophage-specific miR-21–deficient mice (miR-21 cKO) were generated by crossing miR-21–floxed mice (Patrick et al J Clin Invest 2010) with Cx3cr1-Cre tg/0 mice (The Jackson Laboratory Stock no: 025524; RRID:IMSR_JAX:025524; Yona et al Immunity 2013).
Wild animals	No wild animals were used in this study.
Reporting on sex	Only female mice were used.
Field-collected samples	No field-collected samples were used in this study.
Ethics oversight	All animal studies were performed in accordance with relevant guidelines and regulations of the responsible authorities and approval was obtained from the IRB at the Regierung von Oberbayern (ROB-55.2-2532.Vet_02-19-82).

Note that full information on the approval of the study protocol must also be provided in the manuscript.

Flow Cytometry

Plots

Confirm that:

- [x] The axis labels state the marker and fluorochrome used (e.g. CD4-FITC).
- [x] The axis scales are clearly visible. Include numbers along axes only for bottom left plot of group (a 'group' is an analysis of identical markers).
- [x] All plots are contour plots with outliers or pseudocolor plots.
- [x] A numerical value for number of cells or percentage (with statistics) is provided.

Methodology

Sample preparation

Mice:
Prior to harvesting the lungs, bronchoalveolar lavage fluid was collected by sequential (about 5 times) instillation and aspiration of PBS containing 2 mM EDTA through insertion of cannula in the trachea. Lungs were harvested and washed in ice-cold PBS containing 2 mM EDTA and were cut into very small pieces in 3 ml enzyme digestion solution containing Collagenase II (Worthington, Lakewood, NJ), dispase I (Gibco) and DNaseI (Corning, Bedford, USA) followed by incubation at 37°C for 25 minutes. The digestion was stopped by addition of 500 µl of fetal calf serum. Cells were washed in FACS buffer (PBS containing 2 mM EDTA and 0.5% biotin-free bovine serum albumin) and filtered through a 70 µm cell strainer.

Flow cytometry/cell sorting of mice: Cell pellets were then treated with anti-mouse CD16/CD32 (Fc block, diluted 1:50 in FACS buffer) at 4°C for 15 min before being incubated with magnetic microbeads-conjugated anti-CD45 primary antibody at 4°C for 20 min in a shaker. Leukocyte fraction was separated from other cell fractions using AutoMACS (Miltenyi Biotec, Bergish Gladbach, Germany) with the program 'possel'.
Immune cell- enriched fractions from the lung were either stained with antibodies against CD45-FITC, F4/80-PECy7, SIGLECF-PerCP-EFluor710, CD11b-PE and CD24-PE/Dazzle 594 to isolate macrophages, or with antibodies against CD45-FITC, Ly6G-PE/Dazzle 594, CD3-PECy7 and CD19-PECy5 to isolate neutrophils, T cells and B cells. The flow through lung cell suspension enriched for non-immune cell fractions were stained with antibodies against CD45-FITC, CD140a-PECy7, CD105-PE and EPCAM-PE/Dazzle 594 to analyse fibroblast, endothelial and epithelial cells, respectively. Anti-CD45 antibody was used in the non-immune cell fractions to exclude leukocytes.

Analysis of antimiR-21-FAM in pulmonary cells in mice in vivo: Immune cell enriched fractions to assess macrophages: anti-CD45-PECy5 (30-F11, BioLegend), anti-CD206-PE/Dazzle 594 (C068C2, BioLegend), anti-F4/80-PECy7 (BM8, Thermofisher Scientific) and anti-SIGLECF-PE (1RNM44N, Thermofisher Scientific); (2) Immune cell enriched fractions to assess neutrophils, B and T cells: anti-CD3-PECy7(17A2, BioLegend), anti-CD11b-PE (M1/70, BioLegend), anti-CD19-PECy5 (1D3, Thermofisher Scientific) and anti-Ly6G-PE/Dazzle 594 (1A8, BioLegend; (3) CD45-negative fraction: anti-CD45-PECy5 (30-F11, BioLegend), anti-CD105-PE (MJ7/18, Thermofisher Scientific), anti-CD140a-PECy7 (APA5, Thermofisher Scientific), anti-E-cadherin-PerCP/Cy5.5 (DECMA-1, BioLegend). Prior to staining of the cells, Zombie Red (BioLegend) diluted in PBS was used to stain dead cells.

Human:
Digestion of human lungs: After washing, hPCLS were cut into small pieces in HBSS (Merck Sigma Aldrich) digestion buffer containing Collagenase Type IV 575 U/ml (Worthington), DNAse I 0.3mg/ml (Merck Sigma Aldrich), Dispase II 2U/ml (Merck Sigma Aldrich), Elastase 1.5 U/ml (Worthington) and CaCl2 5mM (Merck Sigma Aldrich), and incubated for 45min-1 hour at 37°C. The cells were filtered through a 40 µm cell strainer and the digestion was stopped with EDTA 50mM and FBS 10% V/V (Merck Sigma Aldrich) in DPBS. An erythrocyte lysis for 2-10 min at RT was performed by adding 1 ml Red Blood Lysis buffer (Miltenyi Biotec) to the cells.

Flow cytometry/cell sorting (human): Another filter step followed and the centrifuged cells were resuspended in 40 µl FACS buffer (PBS containing 2 mM EDTA and 0.5% bovine serum albumin) containing 10 µl of FcR Blocking Reagent (Miltenyi Biotec). The cells were blocked for 10 min at 4°C. Then, the cells were incubated with CD45 magnetic beads for 15 min at 4°C and the leukocyte fraction was separated from other cell fractions using AutoMACS (Miltenyi Biotec) with the program 'possel'. Immune cell-enriched fractions from the lung were stained with Zombie Violet dye (BioLegend) and antibodies against CD45-FITC (HI30, BioLegend) and CD206-PECy5 (15-2, Sony Biotechnology) to isolate macrophages. In case of FAM-labelled treated hPCLS, CD45+ fractions and CD45- negative fractions from the lung were stained with CD45-PE (HI30,

BioLegend) and CD206 PE-Cy5 (15-2, Sony Biotechnology) for 30min at 4°. Cells were analysed using Sony SH800 sorter using the 130µm sorting chip.

Instrument	SONY SH800 using a 130µm sorting chip.
Software	SONY SH800 software and FlowJo v 10.7.1.
Cell population abundance	For human slices:<br>A pre-separation for CD45+ and Cd45- cell populations were preformed using MACS. A second round was performed using FACS, so that the highly-enriched macrophage population should not contain any contamination after sorting.<br><br>Mice:<br>The cell type population abundance for the FACS sorted cells are as listed in the Supplementary Figure 2. For the FACS sorted cells, we then confirmed the enrichment of the desired cell population by comparing transcriptome and mirnome profiles of the sorted cells for known cell type markers to the cell surface marker patterns used for flow cytometry.
Gating strategy	Human:<br>Cells were gated by SSC-A/FSC-A. Single cells were identified by FSC-W. Leukocytes were identified by high CD45 expression and non-leukocytes by the absence of CD45 expression. Both fractions (CD45+ and CD45- fraction) were further gated by using CD206.<br><br>Mice:<br>Cells were gated by SSC-A/FSC-A. Single cells were identified by FSC-W. Leukocytes were identified by high CD45 expression and were used to either isolate macrophages or neutrophil/lymphocytes. Macrophages were further gated by CD24- and high expression of F4/80. Macrophages were gated to then identify alveolar macrophages (MP-A) by high SIGLECF or high CD206 expression and low CD11b expression, while interstitial macrophages were identified by low SIGLECF or low CD206 expression and high CD11b expression.<br>Neutrophils within leukocyte population were identified by high Ly6G expression and high CD11b expression. Leukocytes that were negative for Ly6G and CD11b were further subjected to gating to identify T cells (high CD3 and low CD19) and B cells (low CD3 and high CD19).<br><br>Non-leukocytes were identified by low CD45 expression and were further gated using CD105 and EpCAM to identify endothelial cells (high CD105 and EpCAM-) and epithelial cells (CD105- and high EpCAM expression). Cells negative for both CD105 and EpCAM were further gated to identify fibroblasts using CD140a expression.

☒ Tick this box to confirm that a figure exemplifying the gating strategy is provided in the Supplementary Information.
